# Landscape composition and configuration in the central highlands of Ethiopia

**DOI:** 10.1002/ece3.2477

**Published:** 2016-09-25

**Authors:** Terefe Tolessa, Feyera Senbeta, Moges Kidane

**Affiliations:** ^1^ Center for Environment and Development College of Development Studies Addis Ababa University Addis Ababa Ethiopia; ^2^ Department of Natural Resource Management College of Agriculture and Veterinary Science Ambo University Ambo Ethiopia

**Keywords:** ecosystem services, fragmentation, land use and land cover, landscape composition, landscape configuration, metric

## Abstract

Landscape dynamics are common phenomenon in the human‐dominated environments whereby it can be observed that the composition and configuration between landscape elements change over time. This dynamism brings about habitat loss and fragmentation that can greatly alter ecosystem services at patch, class, and landscape levels. We conducted a study to examine composition and configuration of forested landscape in the central highlands of Ethiopia using satellite images of over a period of four decades, and FRAGSTAT raster dataset was used to analyze fragmentation. Our result showed five land use/land cover (LULC) types in the study area. Cultivated land and settlement land increased at the expense of forestland, shrubland, and grassland. Fragmentation analysis showed the number of patches increased for all LULC types, indicating the level of fragmentation and interspersion. Juxtaposition increased for shrubland, grassland, and cultivated lands and decreased for settlement and forestland resulting in the fragmentation and isolation of patches. The study of LULC along with fragmentation at the landscape level can help improve our understanding of the pace at which conversion of landscape elements is happening and the impacts on ecosystem services as studies of LULC are courser in nature and would not show how each land use is reducing in size, proximity and shape among other things that determine ecosystem services. Such type of studies in rural landscapes are very vital to consider appropriate land management policies for the landscape level by taking into account the interaction between each element for sustainable development. We recommend land managers, conservationists, and land owners for observing the roles of each patch in the matrix to maximize the benefits than focusing on a single element.

## 1 Introduction

Landscape structure involves the study of composition and configuration of ecosystems at the landscape level (Mitchell, Bennett, & Andrew, 2013). Composition is mainly concerned with land cover types which are presented at courser scale, whereas configuration refers to fragmentation of habitats reflected at landscape, class, and patch level for specific land use/land cover types, but both occur due to habitat conversion and loss. The spatial arrangement of ecosystems across landscapes is explained by the composition and configuration over period of time. These composition and configuration of ecosystems characterize landscapes as heterogeneous in their nature. Such changes in the composition and configuration are the results of anthropogenic activities (Echeverria, Coomes, Hall, & Newton, 2008; Wu, 2013). Understanding the extent, spatial character and distribution of forest patches within the mosaics of landscapes modified by human activities represent one dimension of theories from island biogeography although fragmentation studies have advanced much more than island biogeography through metapopulation theory and landscape ecology (for example, Erik & Priya, 2003; Laurance, 2008; MacArthur & Wilson, 1967; McGarigal, Cushman, & Ene, 2012).

Anthropogenic habitat loss and fragmentation commonly influence ecosystem services provided by forests, shrubs, and grasslands across landscapes on spatial and temporal scales (Berhane, Totland, & Moe, 2013; Cuke & Srivastava, 2016; Fetene et al., 2016; Laurance, 2008; Midha & Mathur, 2010; Pinto‐Ledezma & Rivero, 2014; Wang & Yang, 2012; Zipperer, Foresman, Walker, & Daniel, 2012). Many studies conducted so far in different parts of the world on various ecosystem types revealed that habitat loss has a greater effect on ecosystem services such as biodiversity conservation among other things than fragmentation (Debuse, King, & House, 2007; Fahrig, 2003; Hillers, Veith, & Rödel, 2008; Peh, Lin, Luke, Foster, & Turner, 2014; Yaacobi, Ziv, & Rosenzweig, 2007). In some instances, the study of habitat loss was not disentangled from habitat fragmentation and the confounding factors are usually reported (McGarigal and Cushman, 2002; Fahrig, 2003). In the case of habitat loss, forest patch is converted to other land uses which change the ecosystem services of a given cover, whereas in fragmentation of landscape, the forest patch reduces in size, changes in shape, increases isolation, and has increased edges at the expense of the interior habitat, and the number of patches increases that can alter the ecosystem services such as biodiversity conservation, pollination, carbon sequestration, and seed dispersal (Çakır, Sivrikaya, & Keleş, 2008; De Marko & Coelho, 2004; Debuse et al., 2007; Hartter & Southworth, 2009; Herrera & Garcia, 2010; Kremen et al., 2007; Li et al., 2007; Putz et al., 2014; Qi, Ye, Zhang, & Yu, 2014; Wang & Yang, 2012), but both are landscape processes.

Habitat loss and fragmentation are nonrandom processes where the conversion of forestland use to agricultural, settlement, and grazing land use is undertaken intentionally by farmers based upon potential productivity of the land for crop production, proximity to roads and urban centers, topography, and drainage (Laurance, 2008). Landscape pattern metrics provide a relative measure of fragmentation, facilitating comparisons between different geographic areas, as well as multitemporal analysis within the same area. The study of fragmentation also depends on the results of change in land cover, and it employs vector or raster data for fragmentation analysis of a given matrix where modified landscapes exist. The degree of fragmentation has been described as a function of patch, class, and landscape metrics described in the Methods section because the best way to quantify the relative importance of habitat loss and fragmentation is to conduct comparative analyses at the landscape scale because a combination of both landscape‐ and patch‐scale variables determines structure and function of ecosystems (Laurance, 2008; Santos‐Filho, Peres, da Silva, & Sanaiotti, 2012). In Ethiopia, several studies have been conducted to understand LULC particularly with reference to deforestation (Feoli, Vuerich, & Zerihun, 2002; Gebrehiwot, Bewket, & Bishop, 2014; Gebrehiwot, Bewket, Gardenas, & Bishop, 2014; Reid et al., 2000; Tsegaye, Moe, Vedeld, & Aynekulu, 2010; Wondrade, Dick, & Tveite, 2014; Zeleke & Hurni, 2001) but to the best knowledge of the authors, no investigation has been carried out on fragmentation analysis combined with LULC.

In this study, we explore landscape composition and configuration and its implication on landscape structure in Jibat Forest because it has been used to infer the spatial and temporal integrity of ecological processes. This assessment identifies how land use changes and fragmentation varies within a rural landscape particularly within a forest boundary that affects patch dynamics and connectivity. We compared land use and land cover changes and fragmentation processes in the study area on temporal scales. The study of landscape composition along with landscape configuration is particularly very useful as fragmentation provides detailed analysis of the changes over time.

We selected Jibat Forest as our study site because it is one of the few remnant forests in a highly modified landscapes in the central highlands of Ethiopia and it is one of the centers of diversity for plant and animal species (Tamrat, 1994; Tesfaye, Fashing, Bekele, Mekonnen, & Atickem, 2013). It is also one of the remnant moist afromontane forests of the country found in the western escarpments servicing as headwater for Gibe River which is serving the country as major source of hydroelectric power generation. In addition, although studies related to vegetation characterization (Tamrat, 1993, 1994) and feeding behavior of Boutourlini's blue monkeys (*Cercopithecus mitis boutourlinii*) were conducted (Tesfaye et al., 2013), analysis of landscape composition and configuration was not conducted and hence, we believe that studying landscape structure is an important aspect to consider in order to understand how the changes in landscape structure can have implication for appropriate management of the resources.

## 2 Materials and Methods

### 2.1 Study area

**Figure 1:**
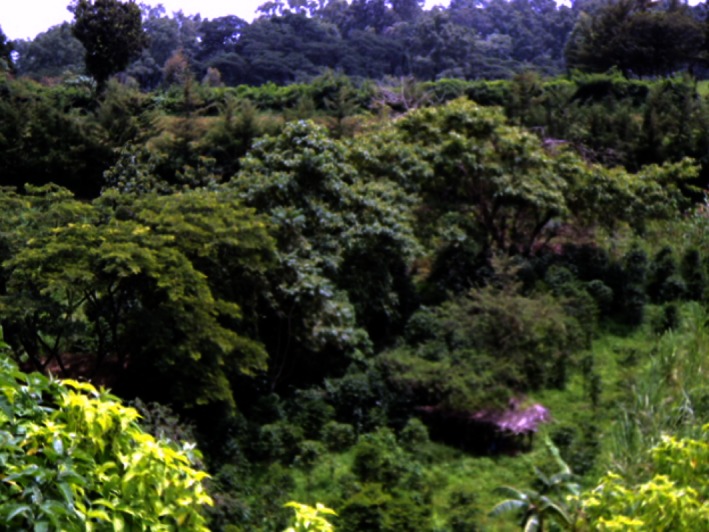
Partial view of forest fragment of the study area

**Figure 2:**
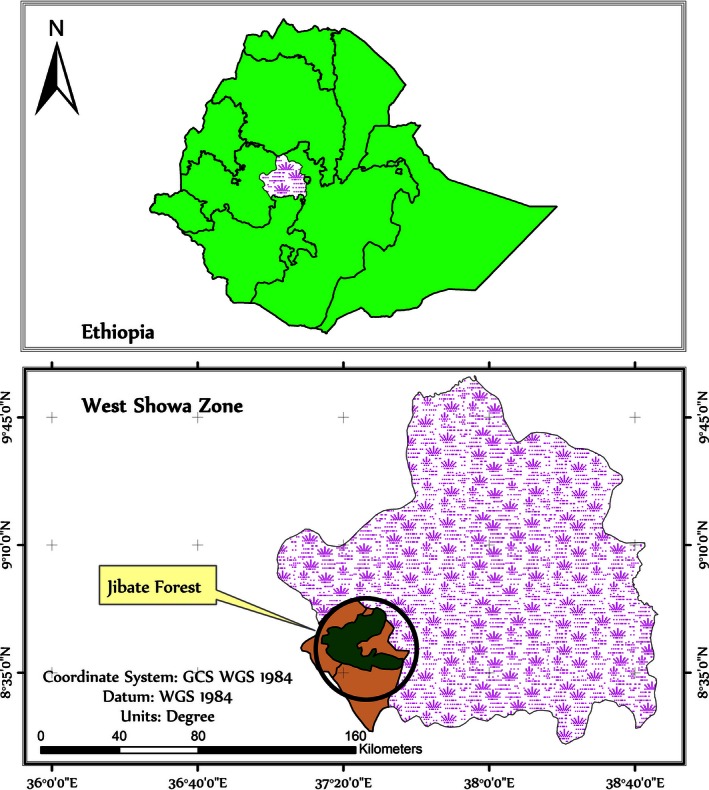
Location of the study area

Jibat Forest is located on the mountain chains of the central highlands of Ethiopia (37°15′–37°30′E; 8°35′–8°50′N) (Figures 1 and 2). It extends from the west to southern portion of the lower altitudes (2,000–3,000 masl) where the forest takes a form of mosaics of landscapes interspersed with farmlands. The forest has been heavily exploited for commercial timber production, agricultural land expansion, and logging by the communities for selling of the wood for small‐scale wood industries (Tamrat, 1993). As a result, the forest did not reach its climax state, rather the forest is regarded as secondary as it is highly disturbed. *Hagenia* and *Rapanea* species which are the characteristics of high‐altitude forests are growing, and the forest is regarded as humid Afromontane forest type (Tamrat, 1993, 1994). On the top of the Mount Jibat, highland bamboo (*Arundinaria alpina*) is dominant and grown extensively along with woody plant species. Through planting of different species of seedlings of commercial trees, there is also wide range of exotic forest plantation particularly *eucalyptus* and *Cuppressus* species, which is used for enrichment planting of logged natural forests.

Forest conversion to agriculture and grazing lands began in the 1970s and has resulted in ongoing fragmentation around the edges of the forest; pioneer tree species such as *Bersama abyssinica* and *Clausena anisata* are the most dominant species (Tesfaye et al., 2013). Livestock currently graze legally both on grasses in recently cleared grazing land near the edge of the forest and on forest shrubs in some peripheral portions of the forest. Illegal tree cutting occurs throughout the forest, although it reaches its highest intensity along the forest edge and in fragmented areas of the forest. The current total area of the forest is estimated to be 38,461 ha (Tesfaye et al., 2013). The total area considered for our study is much higher than the current amount specified because the demarcation processes over the study period were not consistent across the years and the boundary use to change inwards as the forestland was converted to cultivated land through clearing of the vegetation as this method is one way by which farmers obtain the right to cultivated land in Ethiopia. The total area of the forest reported does not include forest patches isolated from the continuous forest due to land use conversion. In this particular study, we tried to incorporate that landscape matrix to take into account fragmentation and land use change processes on spatial and temporal scales as our analysis goes beyond the current boundary of the forest.

Mean annual rainfall was 1,768 mm and mean monthly low and high temperatures were 7.8 and 23.6°C, respectively, for the area between 1997 and 2006. The rainy season occurs from March to October with a peak in rainfall between June and September, and the dry season occurs from November to February (Tesfaye et al., 2013).

### 2.2 Satellite data preprocessing and land use classification

In this study, time series datasets of LULC were produced from multispectral Landsat imagery, which were acquired on four separate years: 1973, 1984, 2000, and 2015. All of the images were clear and nearly free of cloud as it was taken during dry season (Table 1). Prior to interpretation, atmospheric correction and geometrical rectification were performed. The dates selected for processing of LULC were mainly dependent on the availability of the image, important dates in the change of government, and policies related to rural land and agriculture.

**Table 1:** Description of imagery data used for land cover change study in Jibat Forest

Imagery date	Imagery type	Resolution	Path and raw	Source
01/31/1973	Landsat MSS	57 × 57 m	181/54	USGS
02/07/1986	Landsat TM	30 × 30 m	169/54	USGS
01/31/2001	Landsat ETM^+^	30 × 30 m	169/54	USGS
01/26/2015	Landsat OLS	30 × 30 m	169/54	USGS

The image processing and data manipulation were within ArcGIS software. Five land use types classified as settlement land, cropland, shrubland, grassland, and forestland were identified within the study area. Land use/land cover classification signature was prepared for each class; historical training site, image interpretation and personal experience, knowledge of the watershed physical topography were used. Each signature was evaluated by separability test between and within the signature; finally, signature recorded good separability kept; and others are redefined until the signature separability was within an acceptable range.

Ground control points were collected to compute accuracy assessment for the classification year of 2015. The number of GPS points collected was determined using the classification area proportion of the LULC map of 2015. The overall producer's accuracy, overall user's accuracy, and overall Kappa statistics were 85.4%, 89.2%, and 0.84, respectively. These met the recommended values suggested by Janssen and Vander Wel (1994). Thus, these data were available for further study on the level of fragmentation. There are various change detection methods that can be used in ERADS Imagine and other remote sensing software. In this study, the postclassification comparison was employed using separately classified Landsat images and then, three comparisons were made: 1973–1986, 1986–2001, and 2001–2015. To study the changes in land use/land cover between the above year intervals, conversion matrix models are applied. The direction of land use/land cover change between classified images used ERADS Imagine matrixes. During characterization of land cover about five land cover types were identified (Table 2).

**Table 2:** Description of land cover types identified in Jibat Forest

Land cover	Description
Settlement land/Built‐up areas	Land dominated with houses and huts
Cultivated land	Land under cultivation.
Shrubland	Land with >20% bush or shrub cover with <20% tree cover (<5 m in height).
Grazing Land/Grassland	Land under grass cover but highly influenced by grazing and browsing of domestic animals.
Forestland	Land dominated by trees with greater than 80% canopy cover

### 2.3 Measurement of landscape fragmentation

In order to assess landscape fragmentation, we adopted McGarigal et al. (2012) and Smiraglia, Ceccarelli, Bajocco, Perini, and Salvati (2015) landscape metrics (see Table 3). To measure land fragmentation under different land use, fragmentation metrics at landscape levels were selected (Wang & Yang, 2012) depending on the metrics we desire to address because each classes have their own unique characteristics to be described in landscape structure studies. Class‐level metrics such as number of patches, percentage of landscape, edge density, largest patch index, mean patch size, area‐weighted mean shape index, mean Euclidean nearest neighbor distance, interspersion, and juxtaposition and aggregation index were employed to measure the average fragmentation (McGarigal et al., 2012). These fragmentation matrices are the best methods to compare the level of fragmentation of land uses over temporal scales.

**Table 3:** Landscape metrices used in this study area following McGarigal et al. (2012) and Smiraglia et al. (2015)

Acronym	Metric	Description
PLAND	Percentage of landscape	Proportion of the landscape occupied by certain LULC class (0 < PLAND < 100)
NP	Number of patches	Number of patches in the landscape of the same LULC class (*N* ≥ 1)
LPI	Largest patch index	Percentage of the landscape comprised by the largest patch of the corresponding LULC class (0 < LPI < 100)
ED	Edge density	Total length of edge of a certain LULC class per unit area (m/ha). ED ≥ 0, and 0 when there is edge in the landscape
AREA_MN	Mean patch size	Mean area of patches of the same LULC class (m2)
SHAPE_AM	Area‐weighted mean shape index	It measures the complexity of patch shape of a particular LULC class compared to a standard shape (square), by weighting patches according to their size. It equals 1 when all patches are square and increase with complexity of patch shapes.
ENN_MN	Mean Euclidean nearest neighbor distance	Mean of minimum edge‐to‐edge distances to the nearest neighboring patch of the same type of a certain LULC class (m)
IJI	Interspersion and juxtaposition index	Measure of evenness of patch adjacencies equals 100 for even and approaches 0 for uneven adjacencies
AI	Aggregation index	Percentage of neighboring pixel of the same LULC class, based on single‐count method

In this study, computation of the above landscape metrics was performed with FRAGSTATS 4.2.1 (McGarigal et al., 2012) on raster datasets as an input because of its accuracy for calculating fragmentation metrics and the ease of the use of the program on raster datasets (MacLean & Congalton, 2015). The raster datasets were processed in ArcGIS software for use by FRAGSTATS.

## 3 Results

### 3.1 Analysis of land use dynamics

#### 3.1.1 Land use/land cover change

**Figure 3:**
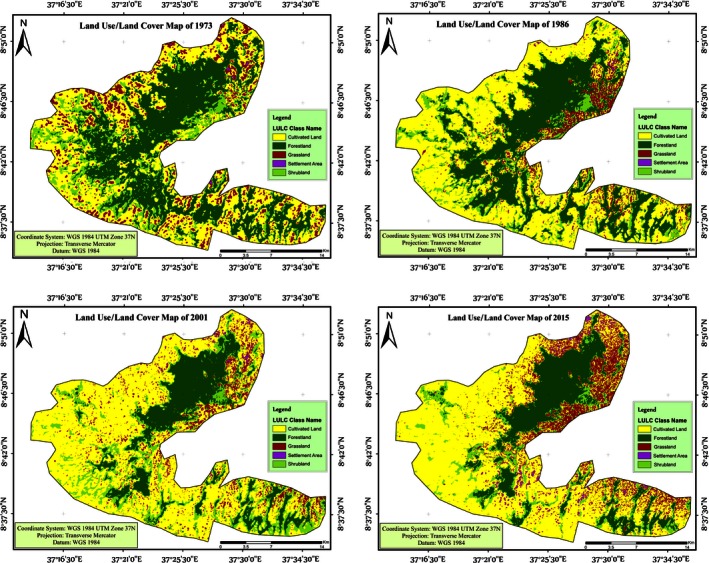
Land use/land cover for four decades (1973–2015)

Land cover classification from the five land use types identified across the four periods indicates the conversion of forestlands and shrublands to agriculture and settlement lands (Table 4; Figure 3). The dominant land use that increased progressively over the study period was agricultural land and settlement land. For example, cultivated land increased by 20.8% from 1973 to 1986, while settlement land increased by 31.1% from 1986 to 2001. On the other hand, forest cover decreased by 14.7% between 1973 and 1986 and by 38.5% between 1986 and 2001. Shrublands were also reduced by 25.9% from 2001 to 2015. During the study period, shrubland was mainly converted to cultivated, settlement, and grasslands. On a major basis, grassland was converted to cultivated land. The greatest net change recorded was for forestland followed by cultivated land.

**Table 4:** Land use/land cover changes of the landscape of the study area, 1973–2015

LULC Class	Absolute area coverage (ha)				Cover change between periods (%)			
	1973	1986	2001	2015	1973–1986	1986–2001	2001–2015	1973–2015
Settlement land	673.92	1378.98	1807.74	3031.02	+10.5	+31.1	+67.67	+349.8
Cultivated land	23489.19	28370.4	34702.3	33560.3	+20.8	+22.3	−3.3	+42.9
Shrubland	10206.3	9869.4	10152.8	7523.55	−3.3	+2.9	−25.9	−26.3
Grassland	5836.86	3894.39	4248.72	6756.39	−33.3	+9.1	+59.0	+15.8
Forestland	22509	19202.1	11803.8	11844.1	−14.7	−38.52	+0.34	−47.4
Total	62715.27	62715.27	62715.27	62715.27				

Forestland lost to cultivated land and shrubland, whereas cultivated land gained from forest land, shrubland, and grassland in the magnitude of their values of conversion, respectively. Settlement area increased by 349.8% during the study period, indicating built‐up rural houses for new couples after marriage.

Of the natural vegetation cover types, forestland and shrubland experienced the lowest persistence, whereas grassland was the most persistent cover type (Table 5). The net change‐to‐persistence ratio was large for forestland (negative), cultivated land (positive), shrubland (negative), settlement land (positive), and grassland (positive), indicating the most dominant trends in the changing landscape (Table 5). Overall, 35910.4 ha (i.e., sum of diagonal elements) of the total landscape remains unchanged (Table 5).

**Table 5:** Land use/land cover transition matrix of the major changes in the landscape (ha), central highlands of Ethiopia, 1973–2015

	To final state (2015)						
	Settlement	Shrubland	Cultivated land	Grassland	Forestland	Total 1973	Loss
From initial state (1973)							
Settlement	**671.3**	0.4	1.1	0.5	0.6	673.9	2.6
Shrubland	416.6	**3279.9**	5160	1010.5	339.4	10206.4	6926.5
Cultivated land	1515.2	130	**18489.9**	3330.2	23.9	23489.2	4999.3
Grassland	410.3	154.8	3253.3	**2003.8**	14.7	5836.9	3833.1
Forestland	17.6	3958.5	6656.1	411.4	**11465.5**	22,509	11043.6
						**35910.4** a	
Total 2015	3031	7523.5	33560.3	6756.4	11844.1		
Gain	2359.7	4243.7	15070.5	4752.6	378.6		
Net change^b^	2357.1	−2682.8	10071.2	919.5	−10665		
Net persistence (NP)^c^	3.51	−0.82	0.54	0.45	−0.93		

Bolded diagonal elements represent proportions of each land use/land cover class that were static (persisted) between 1973 and 2015. The loss column and gain row indicate the proportion of the landscape that experienced gross loss and gain in each class, respectively.

All the figures in the table are in percent except Np, which is a ratio.

a The shaded figure is the sum of diagonals and represents the overall persistence (i.e., the landscape that did not change).

b Net change = gain–loss.

c Np refers to net change‐to‐persistence ratio (i.e., net change/diagonals of each class).

### 3.2 Analysis of the dynamics of landscape metrics

During the study period, there have been changes in the size, number, distance, and spatial distribution of fragments, with different patterns for different land uses at the class level. Cultivated land is the predominant landscape matrix with significant increases for the entire studied variable and study period. One very important fragmentation metric which is vital to note for cultivated land is the number of patches. Across the study period, the number of patches increased which is an area of concern where a piece of land is further divided into several smaller patches by the family. Deforestation was found to be pronounced, and the landscapes dominated by forests became fragmented at the first stage followed by the removal of the fragments through time. Each fragmentation variable indicated below showed a decline in the forest and shrubland followed by an increase in cultivation land and settlement land.

The index of interspersion and juxtaposition (IJI) indicates a decrease in the mixing of patches over time, especially in forests, where it changed from 47.1 in 1973 to 15.6 in 2015, which helps to explain changes in landscape patterns (Table 6). This is evident in 2015, where forests distributed at random in the landscape showing a high level of landscape fragmentation in the study area. The nonlinear change of IJI shows that clumping of individual patches is not following unidirectional changes.

**Table 6:** Representation of spatial pattern at class level for five land uses in four periods of the study (1973, 1986, 2001, and 2015), based on nine landscape metrics for the central highlands of Ethiopia

Year	Landscape metric								
	PLAND	NP	LPI	ED	AREA‐MN	SHAPE‐AM	ENN‐MN	IJI	AI
1973									
Shrubland	13.4	1,763	0.98	30.22	4.7	2.9	144.4	54.5	83.2
Settlement	0.68	327	0.03	2.3	1.3	1.3	448.4	47.6	73.0
Grassland	7.6	876	0.18	16.1	5.4	1.9	210.6	33.0	83.8
Forestland	37.9	471	30.1	24.5	50.2	13.8	186.0	47.1	95.3
Cultivated land	40.5	413	12.6	37.2	61.2	11.3	149.7	82.9	92.9
1986									
Shrubland	14.1	2,324	0.49	37.2	3.8	2.9	109.8	74.9	80.2
Settlement	0.92	773	0.02	4.5	0.74	1.4	294	46.5	63.4
Grassland	4.9	2,039	0.42	17.7	1.5	2.8	149.6	71.1	72.7
Forestland	31.5	481	17.6	19.1	40.9	7.3	163.8	46.2	95.6
Cultivated land	48.6	1,004	14.5	33.4	30.2	8.1	99.7	86.1	94.6
2001									
Shrubland	15.8	1,296	1.4	33.8	7.6	5.8	148.9	57.2	84.0
Settlement	1.8	1,530	0.04	8.7	0.74	1.4	205.7	32.5	64.4
Grassland	4.6	2,808	0.08	19.6	1.0	1.9	146.7	48.5	68.3
Forestland	18.9	542	11.9	12.7	21.8	5.7	189.6	22.1	95.2
Cultivated land	58.9	823	49.7	41.3	44.7	21.9	103	69.9	94.6
2015									
Shrubland	11.3	3,020	0.74	39.32	2.33	5.2	127.03	72.52	73.94
Settlement	3.9	3,071	0.05	20.0	0.79	1.8	128.5	33.2	61.5
Grassland	8.6	5,281	0.77	45.4	1.0	5.5	107.3	45.4	60.0
Forestland	19.2	559	12.8	15.8	21.5	7.2	145.7	15.6	94.0
Cultivated land	57.0	1,627	46.4	72.7	21.9	30.8	74.9	77.9	90.2

PLAND percentage of landscape, NP number of patches, LPI largest patch index, ED edge density, AREA‐MN mean patch size, SHAPE‐AM area‐weighted mean shape index, ENN‐MN mean Euclidean nearest neighbor distance, IJI interspersion and juxtaposition, and AI aggregation index.

#### 3.2.1 Shrubland

Shrubland is essentially found along with forestlands. It is highly influenced by human activities next to forestland where some forest products are regularly collected and livestock graze. It serves as a buffer zone, and secondary growth is taking place but there is also conversion to cultivated land as it is very difficult to obtain land for cultivation by farmers. Number of patches, mean patch size, and percentage of landscape are very dynamic and changed both ways, indicating its fluctuation in the different metrics.

#### 3.2.2 Settlement

Settlement AREA_MN, NP, and SHAPE_AM showed a continuous increase over time. Built‐up patches consolidated around cultivated land, shrubland, and at the edge of the forest (as indicated by the decline of ENN_MN) and expanded into forestlands in a scattered way with changes in the interspersion and juxtaposition index (IJI). Percentage of landscape for settlement is the smallest, but the number of patches is the highest for all years, indicating the small size of the houses relative to other land use and the number of households increased from time to time.

#### 3.2.3 Grassland

Grasslands are decreasing in their size within the study period that can be an indication of the regular conversion of the land use to cultivation. Grasslands are the smallest in mean patch size next to settlement as common grazing areas are further partitioned by communities for cultivation and the recent land certification for use by farmers which grant an exclusive use rights and serving as a base for government tax collection from rural land use.

#### 3.2.4 Forestland

Forest cover decreased progressively throughout the study period with lower AREA_MN and LPI and more complex and less aggregated patches (high AI and lower SHAPE_ AM). The number of patches for forestland did not increase significantly because as patches are formed from the continuous forest, they are gradually converted to cultivated land. Hence, it is not surprising to find a relatively small number of patches despite the highest level of deforestation in the area. Habitat loss is a continuous process that not only results in fragmentation but also subsequent reduction in the size of individual fragments over time which finally results in complete removal of the fragments. The mean nearest neighbor distance (ENN_MN) of forests decreased from 186.0 m in 1973 to 163.8 m in 1986 and increased to 189.6 m in 2001 and then decreased to 145.7 m in 2015. The increase in ENN_MN indicates that large‐sized forest patches tended to become more isolated, while the decrease in ENN_MN may be the result of the overall decrease in large patches. For example, the number of patches with <1 ha was 77.5% in 2015 which shows that it is not only the overall size and number of patches decreased but each patch is also affected by edge with nearly no interior forest. The relatively large‐sized forests are found on the rugged slopes, low soil fertility areas, and mountain chains of Jibat (Table 6).

#### 3.2.5 Cultivated land

For cultivated land, we found the highest class values observed for mean patch size (AREA_MN), aggregation index (AI), and the lowest values observed for mean Euclidean nearest neighbor distance (ENN_MN). In addition, percentage of landscape (PLAND) increased progressively over time except for 2015. Number of patches (NP), edge density (ED), area‐weighted mean shape index (SHAPE_AM), and large patch index (LPI) metrics increased with the exception of 1986, whereas AREA_MN showed the reverse pattern (especially in 2008 and 2015), suggesting that cultivated patches had become more complex and fragmented. This has been due to the fact that farmers own many parcels apart from each other depending on the time of conversion of other land uses for cultivation (Table 6).

### 3.3 Landscape composition and configuration

The land use/land cover analysis showed five classes in the study area. These land use and land cover types changed from one to the other in a nonlinear fashion in which case the coverage was moving forward and backward over time and space. Such compositional variations at the landscape level are characteristically the influence of human activities (Figure 3).

From this land use/land cover matrix, the largest amount of change occurred from forest to cultivated land followed by the conversion of shrubland to cultivated land. The conversion of grassland to cultivated land is also the third highest recorded. Cultivated land was converted to grassland when farmers abandon land as it lost fertility that can be considered a fallow land and on temporary basis it is for grazing because communal grazing lands are becoming scarce in the agropastoral central highlands of Ethiopia. Forestland experienced the greatest conversion to other land uses than any other cover types over the study period although such conversion varies from time to time followed by shrubland and grassland. The conversion of shrub and grassland uses to cultivated land signifies the open access nature of those land uses as perceived by farmers, little/no law enforcement to protect the land uses, and higher demand for crop production through extensification than the use of high level of agricultural technologies such as improved seeds, fertilizers, and weed control mechanisms. Cultivated land was also converted to settlement land as new families are formed, and hence, parents provide their available land for their children.

The landscape configuration showed the dominance of cultivated land in area coverage (higher values of PLAND), interspersion within the landscape (higher value for IJI which represents patch types that are equally adjacent to each other), decreased mean patch size (AREA‐MN) except for the year 2001, increased irregularity (higher values of SHAPE‐AM), increased LPI, and increased number of patches (higher NP). On the other hand, forestland showed the reverse, indicating a decreasing trend with the isolation of each patch, low interspersion, and percentage of landscape decreasing over the study period indicating fragmentation.

Other land use types have shown either a decreasing or increasing trend between cultivated and forestland use types. So, at the landscape level, the dynamic changes between the land uses brought a complex landscape configuration that can play an important role to provide a diversity of ecosystem services if each land uses are properly managed in accordance with the productive capacity taking into account local geographic, edaphic, and socioeconomic factors. In addition, each land use type interacts with each other in such a way that the effects of one on another are very complex.

Each of the metric used to describe fragmentation processes at the landscape determines connectivity between patch types, thereby affecting ecosystem services. So, the percentage landscape is very critical to show the overall impacts of each land use benefit at the landscape level. In our study, it can be generally deduced that the overall benefits of each landscape metric are highly dominated by cultivated land as the largest part of the landscape is occupied.

## 4 Discussion

The current study integrated LULC and landscape metrics to try to unravel landscape composition and configuration. The findings depict how land uses are transformed from one to the other and can be used as a basis for formulating rural development policies which can address sustainable livelihoods by integrating appropriate land management strategies. Our results are in agreement with the findings of Smiraglia et al. (2015). Furthermore, LULC dynamics are courser in depicting land use changes and fragmentation analysis is about detailed synthesis of the dynamics of each land use which can be vital for assessment of the level of fragmentation for each land use over time (Uuemaa, Mander, & Marja, 2013). Habitat loss and fragmentation are affecting ecosystem services by reducing the ecosystem functions provided by important land uses such as forestland, shrubland, and grassland uses. The ecosystem services such as regulating and supporting services are highly influenced by changes in land uses. Several studies conducted in different parts of the world confirmed that land use changes especially deforestation impacted ecosystem services (Costanza et al., 1997, 2014; de Groot et al., 2012; MA, 2005).

### 4.1 Land use/land cover analysis

Subsequent reduction in forestland and shrubland was observed across the study period. This has been evidenced by many studies in Ethiopia (Fetene et al., 2016; Meshesha, Tsunekawa, Tsubo, Ali, & Haregeweyn, 2014; Reid et al., 2000; Tsegaye et al., 2010; Wondrade et al., 2014) and many tropical countries (Lira, Tambosi, Ewers, & Metzger, 2012; Nahuelhual, Carmona, Aguayo, & Echeverria, 2014; Putz et al., 2014). Such conversion of forestland, shrubland, and grasslands was anthropogenic in nature conditioned by socioeconomic, political, and institutional factors (Echeverria et al., 2008; Temesgen et al., 2013). The conversion of one land use to the other is dynamic, and it did not follow a linear pattern over a 40‐year period in the study area. This means that certain land use types such as grassland showed increase and decrease patterns. The rate of conversion of forestland tended to reduce over time particularly in the year 2001–2015. A small amount of increase in forestland was recorded, but the overall reduction is recorded for the whole study period. The small positive increase was also reported by local communities when discussion was made. They indicated that the increment was due to law enforcement by the Oromia forest and wildlife enterprise which took over the forest and started to harvest, plant, and protect the remaining forests.

The conversion of forestland, shrubland, and grassland into settlement and cultivated land can be attributed to population increase, policy incentives of the governments, and market failure to value the ecosystem services of the land uses converted and world economic order which encourages farmers to grow exportable crops to the world market. In the last four decades, the population of the study area and the country increased at higher rate than the economic growth of the country (Meshesha et al., 2014). The population of the study area increased through migration from other parts of the country in search of fertile land to cultivate and higher fertility rate of the indigenous population which is similar to the country as a whole (CSA, 2008; Jacob et al., 2015).

From the number of patches of settlement land, it can be observed that the population had increased in the study area which can be considered as one of the major factors of forestland, shrubland, and grassland use conversion to cultivated land and settlement. The population increased at the rate of 2.6% at the country level and 2.9% in Oromia region of which 87.8% live in rural where the study was carried out (CSA, 2008). Furthermore, government policies over the last four decades have been changed with the change of the regime that contributed to the action taken by farmers to convert land uses. Among these are the land use rights over the imperial, dergue and Ethiopian People Revolutionary Democratic Force (EPRDF) was quite different and hence resulted in how land and land‐related resources were managed by farmers. During the imperial period, land and land‐related resources were owned by the local landlords. The protection, development, and use were vested exclusively by the owners of the resources. During the feudal regime, in Jibat, wood processing sawmill was established privately and the processed product was sold in towns. During the military regime, sawmill was confiscated from private owner and transferred to the government ownership. During EPRDF government (currently), the sawmill was removed and the Oromia forest and wildlife enterprise were established to harvest the logs and sell to the privately owned sawmills in towns and cities. Furthermore, conversion of forests to cultivated land was highest during the military and EPRDF government as they were not able to enforce their policies related to land resource.

Government policies on food self‐sufficiency were also another bottleneck for resource conservation as farmers need to produce more, and they are forced to expand their farms to other land uses due to the low level of technology delivered to farmers as well as the economic capacity of the farmer to afford these technologies. Market‐oriented crop production, a policy of the EPRDF government, is also contributing to land use changes in Ethiopia.

Market failure to value the ecosystem services of forests, shrubs, and grassland relative to cultivated land is another factor resulting in deforestation and fragmentation. In this perspective, natural resource assets will inevitably be misused or exploited until realistic long‐term social and environmental costs are internalized and reflected in market prices. The effort made so far to properly value the services given is limited, and this call for pricing mechanisms for carbon trading mechanisms to be in place for community benefits. In general, the proximate causes such as expansion of cultivated land in response to underlying factors such as policy and institutional factors, demographic factors, and economic factors can have multifaceted effects on land use dynamics. This finding is in agreement with the study of Oestreicher et al. (2014) and Tegegne, Lindnera, Fobissie, and Kanninen (2016) who found that deforestation and degradation are mediated by proximate and underlying factors.

### 4.2 Analysis of the dynamics of landscape metrics

The results of fragmentation analysis indicate a change in forest and shrubland over the study period. Deforestation and forest degradation trajectories were predominant and coincided with increased forest fragmentation due to land use decision followed at national level by government and local scale by farmers themselves in response to social, economic, political, and environmental factors. Farmers make decisions for converting forestlands into cultivated land based on accessibility, soil fertility, topography, and drainage among other factors. Hence, it is not surprising to find fewer patches of forests in inaccessible area. This result is in agreement with Pinto‐Ledezma and Rivero (2014).

This finding is an indication of clear increase in deforestation rates from 1973 to 2015, especially in the periods 1973 and 2001, which is same or greater than deforestation rates reported at the national level or than those of other tropical and subtropical regions of the world (FAO, 2010; Teketay, 2001; Zeleke & Hurni, 2001). Furthermore, the temporal changes of matrix as described in the fragmentation analysis above cannot continue to have similar ecosystem services, rather it changes in accordance with the composition of each matrix within the landscape. The quality of the landscape matrix is very vital in determining ecosystem services at the landscape scale, and each one of the patches within affects the overall benefits (Putz et al., 2014; Santos‐Filho et al., 2012).

#### 4.2.1 Shrubland

Shrublands are at the forefront for conversion to cultivated land and used for cattle grazing as an alternative to grazing land when there is scarcity of grasses especially during dry season. The number of patches and largest patch index show increasing rates of fragmentation, and interspersion indicates isolation of patches. In 2001, the mean patch size is higher than any of the year and this may be due the conversion of forest land to secondary forests through selective cutting, fire, and intensive grazing that can reduce the canopy of the forests. This is one strategy by which farmers gradually covert forests into cultivated land.

#### 4.2.2 Settlement

Settlement patches increased by 89% and percentage of landscape increased by 82.6% from 1973 to 2015. It indicated how many households increased within the study period. Although residential area increased to some extent, the absolute area was small and therefore, it was not the main category to impact ecosystem change in the study area (Li et al., 2007). The small‐sized settlements as depicted with mean patch size are a clear indication of the nature and quality of each house built in rural areas as they are not large enough to accommodate large‐sized families of rural Ethiopia. Each family with the house is overcrowded in a very small room reflecting the economic, social, and environmental conditions that must be modified to improve housing.

#### 4.2.3 Grassland

The higher numbers of patches for grasslands indicate greater fragmentation with small‐sized land left for grazing by livestock. This shows the level of scarcity of grazing land and farmers are nowadays shifting to zero grazing for their livestock by reducing the number and type of herd. In some instances when the fertility of the cultivated land decreased, they leave it as a fallow to regain its productive level while it is serving as grazing land. Such fragmentation is undertaking in grassland because most of the time, it is very easy to convert the land use for cultivation. The degradation and conversion of grasslands to cultivated land and settlement in the study area are similar to Fetene et al's. (2016) findings in Nech Sar Park.

#### 4.2.4 Forestland

Forestland experienced a series of changes on spatial and temporal scale. As observed in the field and studies such as Munsi, Areendran, Ghosh, and Joshi (2010) and Riitters, Wickham, Costanza, and Vogt (2016), forest patches were highly edge‐influenced with a decrease in the interior of the forest. The coverage of the forest decreased in size with the shape become more complex and the number of patches increased which is similar to other findings (Aerts et al., 2016; Soverel, Coops, White, & Wulder, 2010; Tapia‐Armijos, Homeier, Espinosa, Leuschner, & de la Cruz, 2015), although it is not significant because the forest patches isolated from large forests are slowly converted to cultivated land which is similar to the findings of other studies such as Echeverria et al. (2008). Despite this fragmentation, forestlands found in the matrix of landscape provide enormous benefits in terms of biodiversity conservation, source of forest product collection, source of water and microclimate amelioration, carbon sequestration (MacLaren, Buckley, & Hale, 2014; Tadesse, Zavaleta, & Shennan, 2014). In general, habitat loss and habitat fragmentation are the observed scenarios for forestland use, which corroborates the findings of Putz et al. (2014) and Zhai, Cannon, Dai, Zhang, and Xu (2015).

#### 4.2.5 Cultivated land

The fragmentation of cultivated land similar to other land uses in the study area is a common phenomenon in Ethiopian landscapes. Due to land shortage especially in the highlands, where about 70% of the population is living, fragmentation and reduction in cultivated land are certain (Ango, Börjeson, Senbeta, & Hylander, 2014; Bekele & Drake, 2003; Benin & Pender, 2001; Teketay, 2001). Teketay (2001) also indicated that the per capita land holding falls 0.6 ha by the year 2015. Currently, in Ethiopia, cultivated lands are further fragmented based on use rights, fertility, accessibility to their homesteads and infrastructure. In addition, partitioning of existing farmlands among families to newly established households is becoming quite common as new families formed get their share from parents. In general, landscape metrics showed that the composition and configuration of arable land in the landscape was further driven by population increase which was more pronounced during the study period. This situation is seriously undermining productivity of land, labor, and other production factors. The lack of land for cultivation forces family labor especially the young who are economically active to move to other livelihood options such as forest product collection, off‐farm activities, and migration to urban centers.

### 4.3 Land use/land cover analysis and landscape metrics

Combining LULC data with fragmentation analysis improves understanding of the level of landscape transformation, the nature of such changes, and how each land use types did aggregated or dispersed from each other (Gillanders, Coops, Wulder, Gergel, & Nelson, 2008; Riitters et al., 2016; Smiraglia et al., 2015; Uuemaa et al., 2013). The analysis of fragmentation at class level provided detailed information in relation to size of each patch, number of patches, percentage of land use within the landscape, and other important variables that can be useful to understand how the different land uses could be used for optimizing ecosystem services such as biodiversity conservation, pollination, erosion control, protecting cultural landscapes, and hydrological cycles at a landscape level than at patch level which is often very difficult on modified landscapes similar to the study area.

So, although this study is not the first to be carried out in this way, it is the first attempt to try to understand landscape metrics along with LULC analysis and its implication in land management practices in the central parts of Ethiopia where land and land‐related resources are becoming fragmented so that it is difficult to develop the land through the use of soil and water conservation practices (Bekele & Drake, 2003).

## 5 Conclusion

Land use/land cover change detection and fragmentation analysis are useful tools to address the amount and location of change and also provided the ability to compare matrix boundary change over the stated study period by the use of ArcGIS and FRAGSTA software. The interspersion, isolation, and connectivity affect (positive and negative depending on the service required) ecosystem service delivery of patches in our study. The forest boundary has been under a continuous change through habitat loss and fragmentation over time. The forests in the landscape not only are becoming increasingly smaller patches, but increasingly isolated producing both environmental and social implications.

Remote sensing and patch analysis methods can be advantageous in efficiently observing and monitoring land cover changes and fragmentation processes that occur in the landscape boundary across multiple dates at multiple locales. Such studies provide an insight into the processes of changes of land uses between themselves and show the need for appropriate land management policies at landscape level than patch level. Hence, shifting our view of fragmented landscapes toward the full inclusion of landscape matrix in our study is very important to understand how landscapes are composed of different land uses, the dynamics of each matrix on spatial and temporal scale, and the implication of such changes for ecosystem service provision.

In this study, we first analyzed LULC dynamics over four decades and found that settlement land, cultivated land, and grassland increased over four decades. Settlement land consistently increased in the study period, while cultivated land showed an increase (1973–2001) and then decreased afterward. Grassland showed a decrease in cover for the year 1973–1986 drastically than any other land use identified but then increased in the subsequent years. On the other hand, forestland and shrubland decreased by 47.4% and 26.3%, respectively.

Our study indicated the dynamic shifts of land uses across spatial and temporal scales. We found that forestlands, shrublands, and grasslands are at the forefront for conversion to the two major land uses particularly to cultivated lands due to economic, demographic, and policy changes in the study area. In addition, fragmentation of all land uses is a common phenomenon indicating the nature of rural small‐scale framing practices in Ethiopia, which is very difficult to properly manage the land.

We also found that fragmentation of the different land uses was evident, indicating the partitioning of each landscape into smaller patches. One interesting finding we observed in our analysis is the fact that the number of patches for forestland did not increase dramatically. This is mainly because isolated forest patches are slowly converted into cultivated land which is a typical case in our study area. But, as compared to forestland, the number of patches for other land uses increased over the study period. Our finding provided up‐to‐date changes in the level of fragmentation processes. The ongoing land use changes and further fragmentation thereof can have significant effects on the net provision of ecosystem services at the landscape.

## Funding information

No funding information provided.

## Conflict of Interest

None declared.
